# A New Ridge-Type Estimator for the Linear Regression Model: Simulations and Applications

**DOI:** 10.1155/2020/9758378

**Published:** 2020-04-14

**Authors:** B. M. Golam Kibria, Adewale F. Lukman

**Affiliations:** ^1^Department of Mathematics and Statistics, Florida International University, Miami, FL, USA; ^2^Department of Physical Sciences, Landmark University, Omu-Aran, Nigeria; ^3^Institut Henri Poincare Centre Emile Borel, Paris, France

## Abstract

The ridge regression-type (Hoerl and Kennard, 1970) and Liu-type (Liu, 1993) estimators are consistently attractive shrinkage methods to reduce the effects of multicollinearity for both linear and nonlinear regression models. This paper proposes a new estimator to solve the multicollinearity problem for the linear regression model. Theory and simulation results show that, under some conditions, it performs better than both Liu and ridge regression estimators in the smaller MSE sense. Two real-life (chemical and economic) data are analyzed to illustrate the findings of the paper.

## 1. Introduction

To describe the problem, we consider the following linear regression model:

(1) $$\begin{array}{c} y=X\beta +\varepsilon , \end{array}$$

where *y* is an *n* × 1 vector of the response variable, *X* is a known *n* × *p* full rank matrix of predictor or explanatory variables, *β* is an *p* × 1 vector of unknown regression parameters, *ε* is an *n* × 1 vector of errors such that *E*(*ε*)=0, and *V*(*ε*)=*σ*^2^*I*_*n*_, *I*_*n*_ is an *n* × *n* identity matrix. The ordinary least squares estimator (OLS) of *β* in (1) is defined as

(2) $$\begin{array}{c} \hat{\beta }={\left(S\right)}^{-1}X^{\prime }y, \end{array}$$

where *S*=*X*′*X* is the design matrix.

The OLS estimator dominates for a long time until it was proven inefficient when there is multicollinearity among the predictor variables. Multicollinearity is the existence of near-to-strong or strong-linear relationship among the predictor variables. Different authors have developed several estimators as an alternative to the OLS estimator. These include Stein estimator [1], principal component estimator [2], ridge regression estimator [3], contraction estimator [4], modified ridge regression (MRR) estimator [5], and Liu estimator [6]. Also, some authors have developed two-parameter estimators to combat the problem of multicollinearity. The authors include Akdeniz and Kaçiranlar [7]; Özkale and Kaçiranlar [8]; Sakallıoğlu and Kaçıranlar [9]; Yang and Chang [10]; and very recently Roozbeh [11]; Akdeniz and Roozbeh [12]; and Lukman et al. [13, 14], among others.

The objective of this paper is to propose a new one-parameter ridge-type estimator for the regression parameter when the predictor variables of the model are linear or near-to-linearly related. Since we want to compare the performance of the proposed estimator with ridge regression and Liu estimator, we will give a short description of each of them as follows.

### 1.1. Ridge Regression Estimator

Hoerl and Kennard [3] originally proposed the ridge regression estimator. It is one of the most popular methods to solve the multicollinearity problem of the linear regression model. The ridge regression estimator is obtained by minimizing the following objective function:

(3) $$\begin{array}{c} {\left(y-X\beta \right)}^{\prime }\left(y-X\beta \right)+k\left(\beta^{\prime }\beta -c\right), \end{array}$$

with respect to *β*, will yield the normal equations

(4) $$\begin{array}{c} \left(X^{\prime }X+kI_{p}\right)\beta =X^{\prime }y, \end{array}$$

where *k* is the nonnegative constant. The solution to (4) gives the ridge estimator which is defined as

(5) $$\begin{array}{c} \hat{\beta }\left(k\right)={\left(S+kI_{p}\right)}^{-1}X^{\prime }y=W\left(k\right)\hat{\beta }, \end{array}$$

where *S*=*X*′*X*, *W*(*k*)=[*I*_*p*_+*kS*^−1^]^−1^, and *k* is the biasing parameter. Hoerl et al. [15] defined the harmonic-mean version of the biasing parameter for the ridge regression estimator as follows:

(6) $$\begin{array}{c} {\hat{k}}_{HM}=\frac{p{\hat{\sigma }}^{2}}{\sum_{i=1}^{p}\alpha_{i}^{2}}, \end{array}$$

where ${\hat{\sigma }}^{2}=\left(Y^{\prime }Y-\beta^{\prime }X^{\prime }Y\right)/\left(n-p\right)$ is the estimated mean squared error form OLS regression using equation (1) and *α*_*i*_ is *i*th coefficient of *α*=*Q*′*β* and is defined under equation (17). There are a high number of techniques suggested by various authors to estimate the biasing parameters. To mention a few, McDonald and Galarneau [16]; Lawless and Wang [17]; Wichern and Churchill [18]; Kibria [19]; Sakallıoğlu and Kaçıranlar [9]; Lukman and Ayinde [20]; and recently, Saleh et al. [21], among others.

### 1.2. Liu Estimator

The Liu estimator of *β* is obtained by augmenting $d\hat{\beta }=\beta +\varepsilon^{\prime }$ to (1) and then applying the OLS estimator to estimate the parameter. The Liu estimator is obtained to be

(7) $$\begin{array}{c} \hat{\beta }\left(d\right)={\left(S+I_{p}\right)}^{-1}\left(X^{\prime }y+d\hat{\beta }\right)=F\left(d\right)\hat{\beta }, \end{array}$$

where *F*(*d*)=[*S*+*I*_*p*_]^−1^[*S*+*dI*_*p*_]. The biasing parameter *d* for the Liu estimator is defined as follows:

(8) $$\begin{array}{c} {\hat{d}}_{\text{opt}}=1-{\hat{\sigma }}^{2}\left[\frac{\sum_{i=1}^{p}\left(1/\left(\lambda_{i}\left(\lambda_{i}+1\right)\right)\right)}{\sum_{i=1}^{p}\left(\alpha_{i}^{2}/{\left(\lambda_{i}+1\right)}^{2}\right)}\right], \end{array}$$

where *λ*_*i*_ is the *i*th eigenvalue of the *X*′*X* matrix and *α*=*Q*′*β* which is defined under equation (17). If ${\hat{d}}_{\text{opt}}$ is negative, Özkale and Kaçiranlar [8] adopt the following alternative biasing parameter:

(9) $$\begin{array}{c} {\hat{d}}_{\text{alt}}=\min\left[\frac{{\hat{\alpha }}_{i}^{2}}{\left({\hat{\sigma }}^{2}/\lambda_{i}\right)+{\hat{\alpha }}_{i}^{2}}\right], \end{array}$$

where ${\hat{\alpha }}_{i}$ is the *i*th component of ${\hat{\alpha }}_{i}=Q^{\prime }\hat{\beta }$.

For more on the Liu [6] estimator, we refer our readers to Akdeniz and Kaçiranlar [7]; Liu [22]; Alheety and Kibria [23]; Liu [24]; Li and Yang [25]; Kan et al. [26]; and very recently, Farghali [27], among others.

In this article, we propose a new one-parameter estimator in the class of ridge and Liu estimators, which will carry most of the characteristics from both ridge and Liu estimators.

### 1.3. The New One-Parameter Estimator

The proposed estimator is obtained by minimizing the following objective function:

(10) $$\begin{array}{c} {\left(y-X\beta \right)}^{\prime }\left(y-X\beta \right)+k\left[{\left(\beta +\hat{\beta }\right)}^{\prime }\left(\beta +\hat{\beta }\right)-c\right], \end{array}$$

with respect to *β*, will yield the normal equations

(11) $$\begin{array}{c} \left(X^{\prime }X+kI_{p}\right)\beta =X^{\prime }y-k\hat{\beta }, \end{array}$$

where *k* is the nonnegative constant. The solution to (11) gives the new estimator as

(12) $$\begin{array}{c} {\hat{\beta }}_{\text{KL}}={\left(S+kI_{p}\right)}^{-1}\left(S-kI_{p}\right)\hat{\beta }=W\left(k\right)M\left(k\right)\hat{\beta }, \end{array}$$

where *S*=*X*′*X*, *W*(*k*)=[*I*_*p*_+*kS*^−1^]^−1^, and *M*(*k*)=[*I*_*p*_ − *kS*^−1^]. The new proposed estimator will be called the Kibria–Lukman (KL) estimator and denoted by ${\hat{\beta }}_{\text{KL}}$.

#### 1.3.1. Properties of the New Estimator

(13) $$\begin{array}{c} E\left({\hat{\beta }}_{\text{KL}}\right)=W\left(k\right)M\left(k\right)E\left(\hat{\beta }\right)=W\left(k\right)M\left(k\right)\beta . \end{array}$$

The proposed estimator is a biased estimator unless *k* = 0.

(14) $$\begin{array}{c} B\left({\hat{\beta }}_{\text{KL}}\right)=\left[W\left(k\right)M\left(k\right)-I_{p}\right]\beta , \end{array}$$

(15) $$\begin{array}{c} D\left({\hat{\beta }}_{\text{KL}}\right)=\sigma^{2}W\left(k\right)M\left(k\right)S^{-1}M^{\prime }\left(k\right)W^{\prime }\left(k\right), \end{array}$$

and the mean square error matrix (MSEM) is defined as

(16) $$\begin{array}{c} \text{MSEM}\left({\hat{\beta }}_{\text{KL}}\right)=\sigma^{2}W\left(k\right)M\left(k\right)S^{-1}M^{\prime }\left(k\right)W^{\prime }\left(k\right)+\left[W\left(k\right)M\left(k\right)-I_{p}\right]\beta \beta^{\prime }{\left[W\left(k\right)M\left(k\right)-I_{p}\right]}^{\prime }. \end{array}$$

To compare the performance of the four estimators (OLS, RR, Liu, and KL), we rewrite (1) in the canonical form which gives

(17) $$\begin{array}{c} y=Z\alpha +\varepsilon , \end{array}$$

where *Z*=*XQ* and *α*=*Q*′*β*. Here, *Q* is an orthogonal matrix such that *Z'Z* = *QX'XQ* = Λ = diag (*λ*_1_, *λ*_2_,…, *λ*_*p*_). The OLS estimator of *α* is

(18) $$\begin{array}{c} \hat{\alpha }=\Lambda^{-1}Z^{\prime }y, \end{array}$$

(19) $$\begin{array}{c} \text{MSEM}\left(\hat{\alpha }\right)=\sigma^{2}\Lambda^{-1}. \end{array}$$

The ridge estimator (RE) of *α* is

(20) $$\begin{array}{c} \hat{\alpha }\left(k\right)=W\left(k\right)\hat{\alpha }, \end{array}$$

where *W*(*k*)=[*I*_*p*_+*k*Λ^−1^]^−1^ and *k* is the biasing parameter.

(21) $$\begin{array}{c} \text{MSEM}\left(\hat{\alpha }\left(k\right)\right)=\sigma^{2}W\left(k\right)\Lambda^{-1}W\left(k\right)+\left(W\left(k\right)-I_{p}\right)\alpha \alpha {\left(W\left(k\right)-I_{p}\right)}^{\prime }, \end{array}$$

where (*W*(*k*) − *I*_*p*_)=−*k*(Λ+*kI*_*p*_)^−1^.

The Liu estimator of *α* is

(22) $$\begin{array}{c} \hat{\alpha }\left(d\right)={\left(\Lambda +I_{p}\right)}^{-1}\left(Z^{\prime }Y+d\hat{\alpha }\right)=F\left(d\right)\hat{\alpha }, \end{array}$$

where *F*(*d*)=[Λ+*I*_*p*_]^−1^[Λ+*dI*_*p*_].

(23) $$\begin{array}{c} \text{MSEM}\left(\hat{\alpha }\left(d\right)\right)=\sigma^{2}F_{d}\Lambda^{-1}F_{d}+{\left(1-d\right)}^{2}{\left(\Lambda +I\right)}^{-1}\alpha \alpha^{\prime }{\left(\Lambda +I\right)}^{-1}, \end{array}$$

where *F*_*d*_=(Λ+*I*)^−1^(Λ+*dI*).

The proposed one-parameter estimator of *α* is

(24) $$\begin{array}{c} {\hat{\alpha }}_{\text{KL}}={\left(\Lambda +kI_{p}\right)}^{-1}\left(\Lambda -kI_{p}\right)\hat{\alpha }=W\left(k\right)M\left(k\right)\hat{\alpha }, \end{array}$$

where *W*(*k*)=[*I*_*p*_+*k*Λ^−1^]^−1^ and *M*(*k*)=[*I*_*p*_ − *k*Λ^−1^].

The following notations and lemmas are needful to prove the statistical property of ${\hat{\alpha }}_{\text{KL}}$:

**Lemma 1 .**

Let *n* × *n* matrices *M* > 0 and *N* > 0 (or *N* ≥ 0); then, *M* > *N* if and only if *λ*_1_ (*NM*^−1^) < 1, where *λ*_1_ (*NM*^−1^) is the largest eigenvalue of matrix *NM*^−1^ [28].

**Lemma 2 .**

Let *M* be an *n* × *n* positive definite matrix, that is, *M* > 0 and *α* be some vector; then, *M* − *αα*′ ≥ 0 if and only if *α*′*M*^−1^*α* ≤ 1 [29].

**Lemma 3 .**

Let ${\hat{\alpha }}_{i}=A_{i}y\;$, *i* = 1, 2, be two linear estimators of *α*. Suppose that $D=Cov\left({\hat{\alpha }}_{1}\right)-Cov\left({\hat{\alpha }}_{2}\right)>0$, where $Cov\left({\hat{\alpha }}_{i}\right)\;i=1\text{,}2$ denotes the covariance matrix of ${\hat{\alpha }}_{i}$ and $b_{i}=Bias\left({\hat{\alpha }}_{i}\right)=\left(A_{i}X-I\right)\alpha ,\;i=1,2$. Consequently,

(25) $$\begin{array}{c} \Delta \left({\hat{\alpha }}_{1}-{\hat{\alpha }}_{2}\right)=\text{MSEM}\left({\hat{\alpha }}_{1}\right)-\text{MSEM}\left({\hat{\alpha }}_{2}\right)=\sigma^{2}D+b_{1}b_{2}^{\prime }-b_{2}b_{2}^{\prime }>0 \end{array}$$

if and only if *b*_2_′[*σ*^2^*D*+*b*_1_*b*_1_′]^−1^*b*_2_ < 1, where $\text{MSEM}\left({\hat{\alpha }}_{i}\right)=Cov\left({\hat{\alpha }}_{i}\right)+b_{i}b_{i}^{\prime }$ [30].

The other parts of this article are as follows. The theoretical comparison among the estimators and estimation of the biasing parameters are given in Section 2. A simulation study has been constructed in Section 3. We conducted two numerical examples in Section 4. This paper ends up with concluding remarks in Section 5.

## 2. Comparison among the Estimators

### 2.1. Comparison between $\hat{\alpha }$ and ${\hat{\alpha }}_{\text{KL}}$

The difference between $\text{MSEM}\left(\hat{\alpha }\right)$ and $\text{MSEM}\left({\hat{\alpha }}_{\text{KL}}\right)$ is

(26) $$\begin{array}{c} \text{MSEM}\left[\hat{\alpha }\right]-\text{MSEM}\left[{\hat{\alpha }}_{\text{KL}}\right]=\sigma^{2}\Lambda^{-1}-\sigma^{2}W\left(k\right)M\left(k\right)\Lambda^{-1}M^{\prime }\left(k\right)W^{\prime }\left(k\right)\\ -\left[W\left(k\right)M\left(k\right)-I_{p}\right]\alpha \alpha^{\prime }{\left[W\left(k\right)M\left(k\right)-I_{p}\right]}^{\prime }. \end{array}$$

We have the following theorem.

**Theorem 1 .**

If *k* *>* 0, estimator ${\hat{\alpha }}_{\text{KL}}$ is superior to estimator $\hat{\alpha }$ using the MSEM criterion, that is, $\text{MSEM}\left[\hat{\alpha }\right]-\text{MSEM}\left[{\hat{\alpha }}_{\text{KL}}\right]>0$ if and only if

(27) $$\begin{array}{c} \alpha^{\prime }{\left[W\left(k\right)M\left(k\right)-I_{p}\right]}^{\prime }\left[\sigma^{2}\left(\Lambda^{-1}-W\left(k\right)M\left(k\right)\Lambda^{-1}M^{\prime }\left(k\right)W{\left(k\right)}_{k}\right)\right]\left[W\left(k\right)M\left(k\right)-I_{p}\right]\alpha <1. \end{array}$$

**Proof**

The difference between (15) and (19) is

(28) $$\begin{array}{c} D\left(\hat{\alpha }\right)-D\left({\hat{\alpha }}_{\text{KL}}\right)=\sigma^{2}\left(\Lambda^{-1}-W\left(k\right)M\left(k\right)\Lambda^{-1}M^{\prime }\left(k\right)W^{\prime }\left(k\right)\right)\\ =\sigma^{2}\text{diag}{\left\{\frac{1}{\lambda_{i}}-\frac{{\left(\lambda_{i}-k\right)}^{2}}{\lambda_{i}{\left(\lambda_{i}+k\right)}^{2}}\right\}}_{i=1}^{p}, \end{array}$$

where Λ^−1^ − *W*(*k*)*M*(*k*)Λ^−1^*M*′(*k*)*W*′(*k*) will be positive definite (pd) if and only if (*λ*_*i*_+*k*)^2^ − (*λ*_*i*_ − *k*)^2^ > 0. We observed that, for *k* > 0, (*λ*_*i*_+*k*)^2^ − (*λ*_*i*_ − *k*)^2^=4*λ*_*i*_*k* > 0.

Consequently, Λ^−1^ − *W*(*k*)*M*(*k*)Λ^−1^*M*′(*k*)*W*′(*k*) is pd.

### 2.2. Comparison between $\hat{\alpha }\left(k\right)$ and ${\hat{\alpha }}_{\text{KL}}$

The difference between $\text{MSEM}\left(\hat{\alpha }\left(k\right)\right)$ and $\text{MSEM}\left({\hat{\alpha }}_{\text{KL}}\right)$ is

(29) $$\begin{array}{c} \text{MSEM}\left[\hat{\alpha }\left(k\right)\right]-\text{MSEM}\left[{\hat{\alpha }}_{k}\right]=\sigma^{2}W\left(k\right)\Lambda^{-1}W\left(k\right)-\sigma^{2}W\left(k\right)M\left(k\right)\Lambda^{-1}M^{\prime }\left(k\right)W\left(k\right)\\ +\left(W\left(k\right)-I_{p}\right)\alpha \alpha {\left(W\left(k\right)-I_{p}\right)}^{\prime }-\left[W\left(k\right)M\left(k\right)-I_{p}\right]\alpha \alpha^{\prime }{\left[W\left(k\right)M\left(k\right)-I_{p}\right]}^{\prime }. \end{array}$$

**Theorem 2 .**

When *λ*_max_(*HG*^−1^) < 1, estimator ${\hat{\alpha }}_{KL}$ is superior to $\hat{\alpha }\left(k\right)$ in the MSEM sense if and only if

(30) $$\begin{array}{c} \alpha^{\prime }{\left[W\left(k\right)M\left(k\right)-I_{p}\right]}^{\prime }\left[\left.V_{1}+\left(W\left(k\right)-I_{p}\right)\alpha \alpha^{\prime }\left(W\left(k\right)-I_{p}\right)\right)\right]\left[W\left(k\right)M\left(k\right)-I_{p}\right]\alpha . \end{array}$$

(31) $$\begin{array}{c} \lambda_{\max}\left(HG^{-1}\right)<1, \end{array}$$

where

(32) $$\begin{array}{c} V_{1}=\sigma^{2}W\left(k\right)\Lambda^{-1}W\left(k\right)-\sigma^{2}W\left(k\right)M\left(k\right)\Lambda^{-1}M^{\prime }\left(k\right)W\left(k\right),\\ H=2W\left(k\right),\\ G=kW\left(k\right)\Lambda^{-1}W\left(k\right). \end{array}$$

**Proof**

Using the dispersion matrix difference,

(33) $$\begin{array}{c} V_{1}=\sigma^{2}W\left(k\right)\Lambda^{-1}W\left(k\right)-\sigma^{2}W\left(k\right)M\left(k\right)\Lambda^{-1}M^{\prime }\left(k\right)W\left(k\right)\\ =\sigma^{2}k\Lambda^{-1}\left(\Lambda W\left(k\right)\Lambda^{-1}W\left(k\right)+\Lambda W\left(k\right)\Lambda^{-1}W\left(k\right)-kW\left(k\right)\Lambda^{-1}W\left(k\right)\right)\Lambda^{-1}\\ =\sigma^{2}W\left(k\right)\Lambda^{-1}W\left(k\right)-\sigma^{2}W\left(k\right)\left[I_{p}-k\Lambda^{-1}\right]\Lambda^{-1}\left[I_{p}-k\Lambda^{-1}\right]W\left(k\right)\\ =\sigma^{2}k\Lambda^{-1}\left(G-H\right)\Lambda^{-1}. \end{array}$$

It is obvious that, for *k* > 0, *G* > 0 and *H* > 0. According to Lemma 1, it is clear that *G*-*H* > 0 if and only if *HG*^−1^ < 1, where *λ*_max_(*HG*^−1^) < 1 is the maximum eigenvalue of the matrix *HG*^−1^. Consequently, *V*_1_ is pd.

### 2.3. Comparison between $\hat{\alpha }\left(d\right)$ and ${\hat{\alpha }}_{\text{KL}}$

The difference between $\text{MSEM}\left(\hat{\alpha }\left(d\right)\right)$ and $\text{MSEM}\left({\hat{\alpha }}_{\text{KL}}\right)$ is

(34) $$\begin{array}{c} \text{MSEM}\left[\hat{\alpha }\right]-\text{MSEM}\left[{\hat{\alpha }}_{k}\right]=\sigma^{2}F_{d}\Lambda^{-1}F_{d}-\sigma^{2}W\left(k\right)M\left(k\right)\Lambda^{-1}M^{\prime }\left(k\right)W^{\prime }\left(k\right)\\ +{\left(1-d\right)}^{2}{\left(\Lambda +I\right)}^{-1}\alpha \alpha^{\prime }{\left(\Lambda +I\right)}^{-1}-\left[W\left(k\right)M\left(k\right)-I_{p}\right]\alpha \alpha^{\prime }{\left[W\left(k\right)M\left(k\right)-I_{p}\right]}^{\prime }. \end{array}$$

We have the following theorem.

**Theorem 3 .**

If *k* *>* 0 and 0 < *d* < 1, estimator ${\hat{\alpha }}_{\text{KL}}$ is superior to estimator $\hat{\alpha }\left(d\right)$ using the MSEM criterion, that is, $\text{MSEM}\left(\hat{\alpha }\left(d\right)\right)-\text{MSEM}\left({\hat{\alpha }}_{\text{KL}}\right)>0$ if and only if

(35) $$\begin{array}{c} \alpha^{\prime }{\left[W\left(k\right)M\left(k\right)-I_{p}\right]}^{\prime }\left[V_{2}+{\left(1-d\right)}^{2}{\left(\Lambda +I\right)}^{-1}\alpha \alpha^{\prime }{\left(\Lambda +I\right)}^{-1}\right]\left[W\left(k\right)M\left(k\right)-I_{p}\right]\alpha <1, \end{array}$$

where *V*_2_=*σ*^2^*F*_*d*_Λ^−1^*F*_*d*_ − *σ*^2^*W*(*k*)*M*(*k*)Λ^.−1^*M*′(*k*)*W*(*k*).

**Proof**

Using the difference between the dispersion matrix,

(36) $$\begin{array}{c} V_{2}=\sigma^{2}F_{d}\Lambda^{-1}F_{d}-\sigma^{2}W\left(k\right)M\left(k\right)\Lambda^{.-1}M^{\prime }\left(k\right)W\left(k\right)\\ =\sigma^{2}\left(F_{d}\Lambda^{-1}F_{d}-W\left(k\right)M\left(k\right)\Lambda^{.-1}M^{\prime }\left(k\right)W\left(k\right)\right)\\ =\sigma^{2}{\left[\Lambda +I_{p}\right]}^{-1}\left[\Lambda +dI_{p}\right]\Lambda^{-1}{\left[\Lambda +I_{p}\right]}^{-1}\left[\Lambda +dI_{p}\right]-\Lambda {\left(\Lambda +k\right)}^{-1}\Lambda^{.-1}\\ \left(\Lambda -k\right)\Lambda^{.-1}\Lambda^{.-1}\left(\Lambda -k\right)\Lambda {\left(\Lambda +k\right)}^{-1}, \end{array}$$

where *W*(*k*)=[*I*_*p*_+*k*Λ^−1^]^−1^=Λ(Λ+*k*)^−1^ and *M*(*k*)=[*I*_*p*_ − *k*Λ^−1^]=Λ^−1^(Λ − *k*)

(37) $$\begin{array}{c} =\sigma^{2}\text{diag}{\left\{\frac{{\left(\lambda_{i}+d\right)}^{2}}{\lambda_{i}{\left(\lambda_{i}+1\right)}^{2}}-\frac{{\left(\lambda_{i}-k\right)}^{2}}{\lambda_{i}{\left(\lambda_{i}+k\right)}^{2}}\right\}}_{i=1}^{p}. \end{array}$$

We observed that *F*_*d*_Λ^−1^*F*_*d*_ − *W*(*k*)*M*(*k*)Λ^.−1^*M*′(*k*)*W*(*k*) is pd if and only if (*λ*_*i*_+*d*)^2^(*λ*_*i*_+*k*)^2^ − (*λ*_*i*_ − *k*)^2^(*λ*_*i*_+1)^2^ > 0 or (*λ*_*i*_+*d*)(*λ*_*i*_+*k*) − (*λ*_*i*_ − *k*)(*λ*_*i*_+1) > 0. Obviously for *k* *>* 0 and 0 < *d* < 1, (*λ*_*i*_+*d*)(*λ*_*i*_+*k*) − (*λ*_*i*_ − *k*)(*λ*_*i*_+1)=*k*(2*λ*+*d*+1)+*λ*(*d* − 1) > 0. Consequently, *F*_*d*_Λ^−1^*F*_*d*_ − *W*(*k*)*M*(*k*)Λ^.−1^*M*′(*k*)*W*(*k*) is pd.

### 2.4. Determination of Parameter *k*

There is a need to estimate the parameter of the new estimator for practical use. The ridge biasing parameter and the Liu shrinkage parameter were determined by both Hoerl and Kennard [3] and Liu [6], respectively. Different authors have developed other estimators of these ridge parameters. To mention a few, these include Hoerl et al. [15]; Kibria [19]; Kibria and Banik [31]; and Lukman and Ayinde [20], among others. The optimal value of *k* is the one that minimizes

(38) $$\begin{array}{c} \text{MSEM}\left({\hat{\beta }}_{\text{KL}}\right)=\sigma^{2}W\left(k\right)M\left(k\right)S^{-1}M^{\prime }\left(k\right)W^{\prime }\left(k\right)+\left[W\left(k\right)M\left(k\right)-I_{p}\right]\beta \beta^{\prime }{\left[W\left(k\right)M\left(k\right)-I_{p}\right]}^{\prime },\\ p\left(k\right)=\text{MSEM}\left[{\hat{\alpha }}_{\text{KL}}\right]=tr\left[\text{MSEM}\left({\hat{\alpha }}_{\text{KL}}\right)\right],\\ p\left(k\right)=\sigma^{2}\sum_{i=1}^{p}\frac{{\left(\lambda_{i}-k\right)}^{2}}{\lambda_{i}{\left(\lambda_{i}+k\right)}^{2}}+4k^{2}\sum_{i=1}^{p}\frac{\alpha_{i}^{2}}{{\left(\lambda_{i}+k\right)}^{2}}. \end{array}$$

Differentiating *m*(*k*, *d*) with respect to *k* gives and setting (∂*p*(*k*)/∂*k*)=0, we obtain

(39) $$\begin{array}{c} k=\frac{\sigma^{2}}{2\alpha_{i}^{2}+\left(\sigma^{2}/\lambda_{i}\right)}. \end{array}$$

The optimal value of *k* in (39) depends on the unknown parameter *σ*^2^ and *α*^2^. These two estimators are replaced with their unbiased estimate. Consequently, we have

(40) $$\begin{array}{c} \hat{k}=\frac{{\hat{\sigma }}^{2}}{2{\hat{\alpha }}_{i}^{2}+\left({\hat{\sigma }}^{2}/\lambda_{i}\right)}. \end{array}$$

Following Hoerl et al. [15], the harmonic-mean version of (40) is defined as

(41) $$\begin{array}{c} {\hat{k}}_{\text{HMN}}=\frac{p{\hat{\sigma }}^{2}}{\sum_{i=1}^{p}\left[2{\hat{\alpha }}_{i}^{2}+\left({\hat{\sigma }}^{2}/\lambda_{i}\right)\right]}. \end{array}$$

According to Özkale and Kaçiranlar [8], the minimum version of (41) is defined as

(42) $$\begin{array}{c} {\hat{k}}_{\min}=\min\left[\frac{{\hat{\sigma }}^{2}}{2{\hat{\alpha }}_{i}^{2}+\left({\hat{\sigma }}^{2}/\lambda_{i}\right)}\right]. \end{array}$$

## 3. Simulation Study

Since theoretical comparisons among the estimators, ridge regression, Liu and KL in Section 2 give the conditional dominance among the estimators, a simulation study has been conducted using the R 3.4.1 programming languages to see a better picture about the performance of the estimators.

### 3.1. Simulation Technique

The design of the simulation study depends on factors that are expected to affect the properties of the estimator under investigation and the criteria being used to judge the results. Since the degree of collinearity among the explanatory variable is of central importance, following Gibbons [32] and Kibria [19], we generated the explanatory variables using the following equation:

(43) $$\begin{array}{c} x_{ij}={\left(1-\rho^{2}\right)}^{1/2}z_{ij}+\rho z_{i,p+1},\;i=1,2,\ldots ,n,\;j=1,2,3,\ldots ,p, \end{array}$$

where *z*_*ij*_ are independent standard normal pseudo-random numbers and *ρ* represents the correlation between any two explanatory variables. We consider *p*=3 and 7 in the simulation. These variables are standardized so that *X*′*X* and *X*′*y* are in correlation forms. The *n* observations for the dependent variable *y* are determined by the following equation:

(44) $$\begin{array}{c} y_{i}=\beta_{0}+\beta_{1}x_{i1}+\beta_{2}x_{i2}+\beta_{3}x_{i3}+\cdots +\beta_{p}x_{ip}+e_{i},\;i=1,2,\ldots ,n, \end{array}$$

where *e*_*i*_ are i.i.d *N* (0, *σ*^2^), and without loss of any generality, we will assume zero intercept for the model in (44). The values of *β* are chosen such that *β*′*β* = 1 [33]. Since our main objective is to compare the performance of the proposed estimator with ridge regression and Liu estimators, we consider *k* *=* *d* = 0.1, 0.2,…, 1. We have restricted *k* between 0 and 1 as Wichern and Churchill [18] have found that the ridge regression estimator is better than the OLS when *k* is between 0 and 1. Kan et al. [26] also suggested a smaller value of *k* (less than 1) is better. Simulation studies are repeated 1,000 times for the sample sizes *n* = 30 and 100 and *σ*^2^ = 1, 25, and 100. For each replicate, we compute the mean square error (MSE) of the estimators by using the following equation:

(45) $$\begin{array}{c} \text{MSE}\left(\alpha^{*}\right)=\frac{1}{1000}\sum_{i=1}^{1000}{\left(\alpha^{*}-\alpha \right)}^{\prime }\left(\alpha^{*}-\alpha \right), \end{array}$$

where *α*^*∗*^ would be any of the estimators (OLS, ridge, Liu, or KL). Smaller MSE of the estimators will be considered the best one.

**Figure 1:**
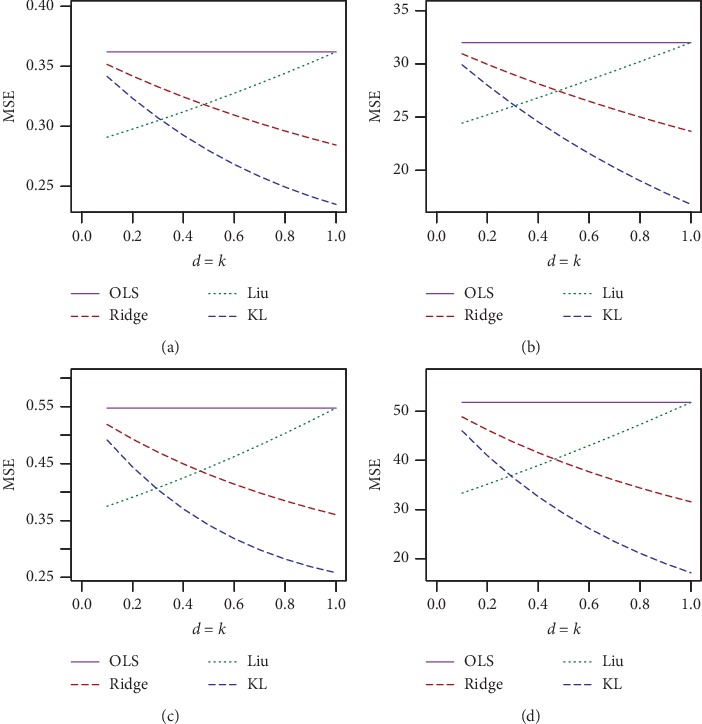
Estimated MSEs for *n* = 30. Sigma = 1, 10, rho = 0.70, 0.80 and different values of *k* = *d*. (a) *n* = 30, *p*=3, sigma = 1, and rho = 0.70. (b) *n* = 30, *p*=3, sigma = 10, and rho = 0.70. (c) *n* = 30, *p*=3, sigma = 1, and rho = 0.80. (d) *n* = 30, *p*=3, sigma = 10, and rho = 0.80.

**Figure 2:**
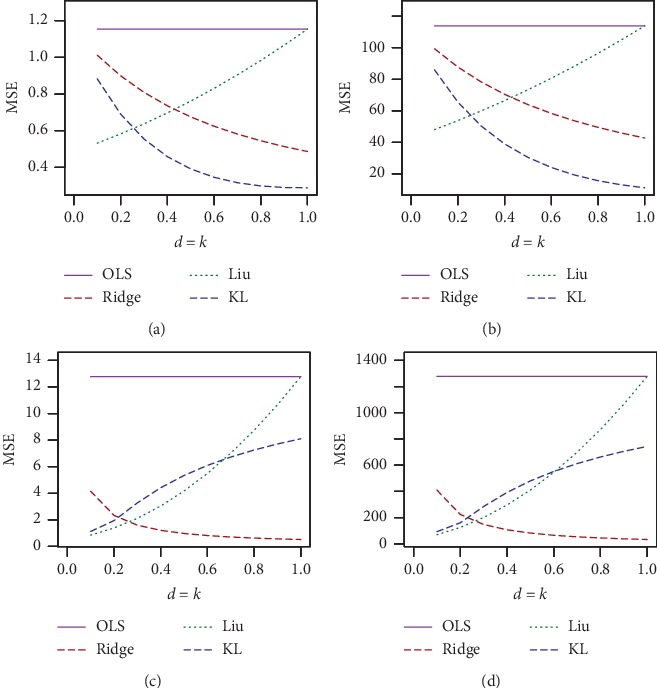
Estimated MSEs for *n* = 30, sigma = 1, 10, rho = 0.90, 0.99, and different values of *k* = *d*. (a) *n* = 30, *p*=3, sigma = 1, and rho = 0.90. (b) *n* = 30, *p*=3, sigma = 10, and rho = 0.90. (c) *n* = 30, *p*=3, sigma = 1, and rho = 0.99. (d) *n* = 30, *p*=3, sigma = 10, and rho = 0.99.

**Figure 3:**
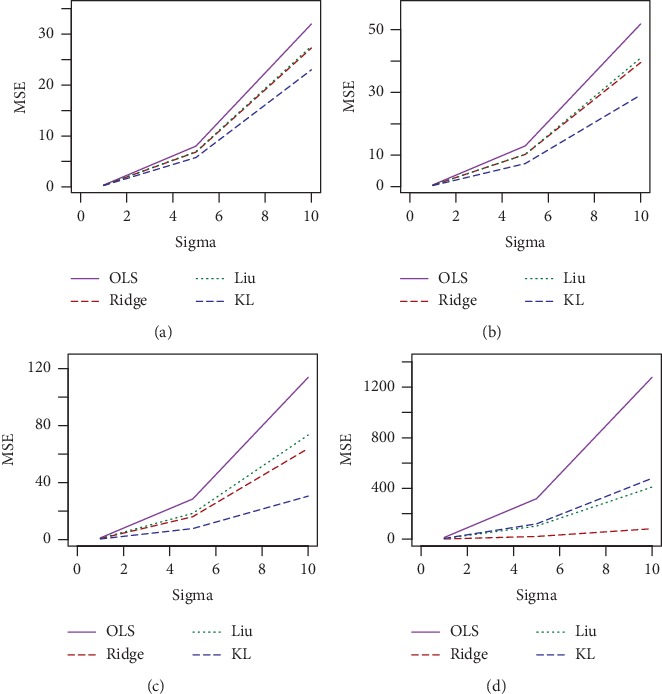
Estimated MSEs for *n* = 30, *d* = 0.5, and different values of rho and sigma. (a) *n* = 30, *p*=3, *d* = 0.5, and rho = 0.70. (b) *n* = 30, *p*=3, *d* = 0.5, and rho = 0.80. (c) *n* = 30, *p*=3, *d* = 0.5, and rho = 0.90. (d) *n* = 30, *p*=3, *d* = 0.5, and rho = 0.99.

**Figure 4:**
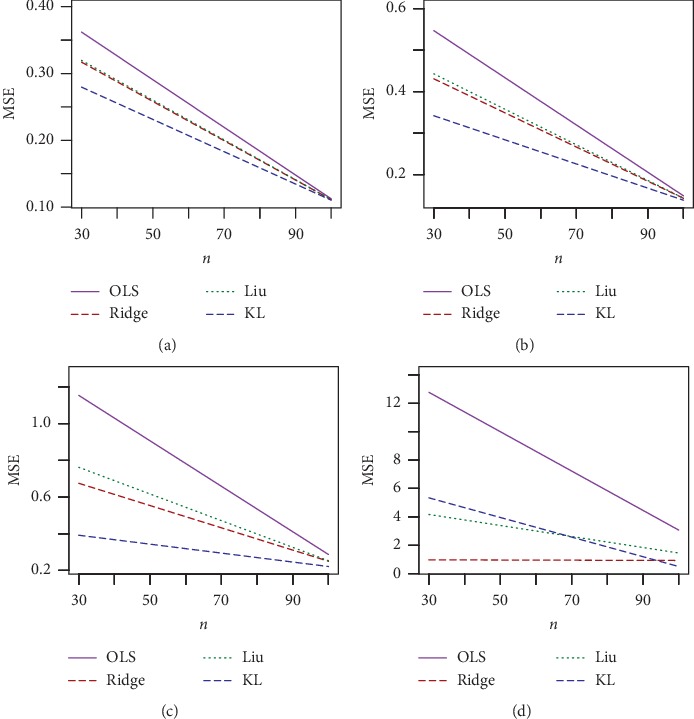
Estimated MSEs for sigma = 1, *p*=3, and different values of rho and sample size. (a)*p*=3, sigma = 1, *d* = 0.5, and rho = 0.70. (b)*p*=3, sigma = 1, *d* = 0.5, and rho = 0.80. (c)*p*=3, sigma = 1, *d* = 0.5, and rho = 0.90. (d)*p*=3, sigma = 1, *d* = 0.5, and rho = 0.99.

**Figure 5:**
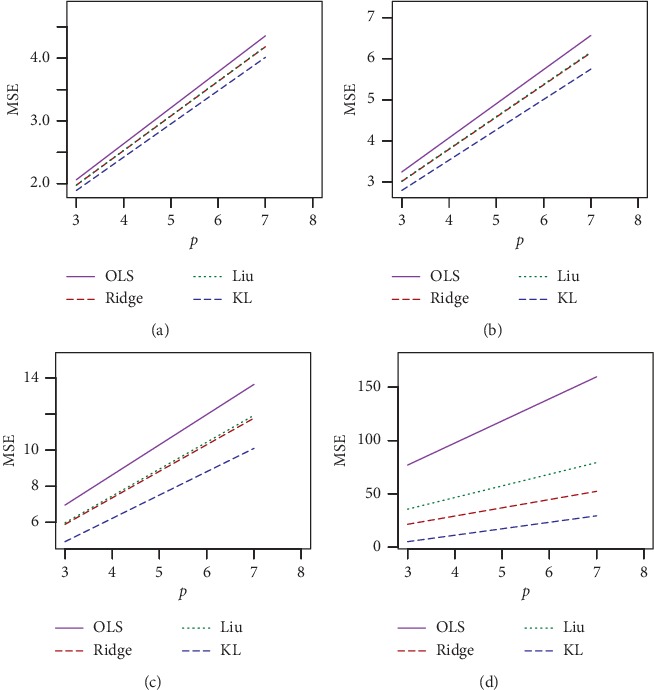
Estimated MSEs for *n* = 100, *d* = 0.5, sigma = 5, and different values of rho and *p*. (a) *n* = 100, sigma = 5, *d* = 0.5, and rho = 0.70. (b) *n* = 100, sigma = 5, *d* = 0.5, and rho = 0.80. (c) *n* = 100, sigma = 5, *d* = 0.5, and rho = 0.90. (d) *n* = 100, sigma = 5, *d* = 0.5, and rho = 0.99.

**Figure 6:**
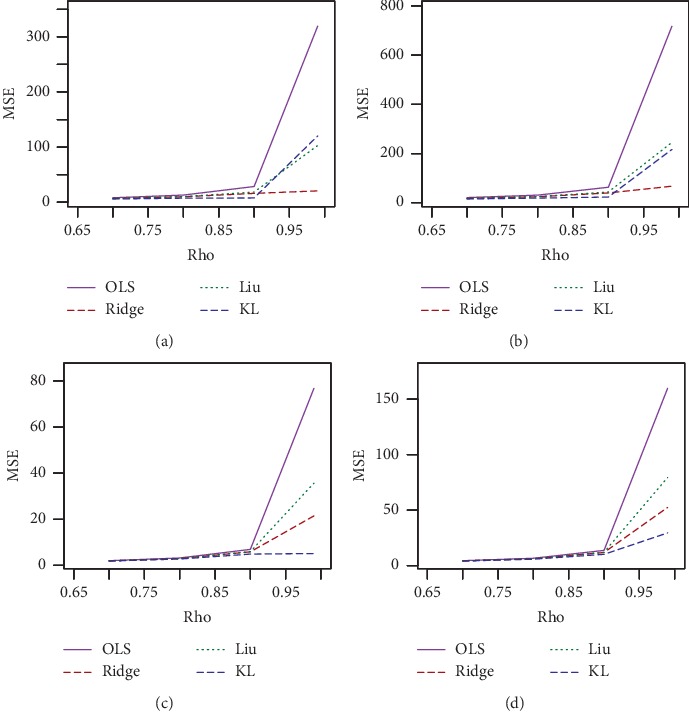
Estimated MSEs for *n* = 100, *p*=3, 7, *d* = 0.5, sigma = 5, and different values of rho. (a) *n* = 30, *p*=3, sigma = 5, and *d* = 0.5. (b) *n* = 30, *p*=7, sigma = 5, and *d* = 0.5. (c) *n* = 100, *p*=3, sigma = 5, and *d* = 0.5. (d) *n* = 100, *p*=7, sigma = 5, and *d* = 0.5.

The simulated results for *n* = 30, *p*=3, and *ρ* = 0.70, 0.80 and *ρ* = 0.90, 0.99 are presented in Tables 1 and 2, respectively, and for *n* = 100, *p*=3, and *ρ* = 0.7, 0.80 and *ρ* = 0.90, 0.99 are presented in Tables 3 and 4, respectively. The corresponding simulated results for *n* = 30, 100 and *p*=7 are presented in Tables 5–8. For a better visualization, we have plotted MSE vs. *d* for *n* = 30, *σ* = 10, and *ρ* = 0.70, 0.90, and 0.99 in Figures 1–3, respectively. We also plotted MSE vs *σ* for *n* = 30, *d* = .50, and *ρ* = 0.90 and 0.99, which is presented in Figures 4 and 5, respectively. Finally, to see the effect of sample size on MSE, we plotted MSE vs. sample size for *d* = 0.5 and *ρ* = 0.90 and presented in Figure 6.

### 3.2. Simulation Results and Discussion

From Tables 1–8 and Figures 1–6, it appears that, as the values of *σ* increase, the MSE values also increase (Figure 3), while the sample size increases as the MSE values decrease (Figure 4). Ridge, Liu, and proposed KL estimators uniformly dominate the ordinary least squares (OLS) estimator. In general, from these tables, an increase in the levels of multicollinearity and the number of explanatory variables increase the estimated MSE values of the estimators. The figures consistently show that the OLS estimator performs worst when there is multicollinearity. From Figures 1–6 and simulation Tables 1–8, it clearly indicated that, for *ρ*=0.90 or less, the proposed estimator uniformly dominates the ridge regression estimator, while Liu performed much better than both proposed and ridge estimators for small *d*, say 0.3 or less. When *ρ*=0.99, the ridge regression performs the best for higher *k*, while the proposed estimator performs the best for say *k* (say 0.3 or less). When *d* = *k* = 0.5 and *ρ*=0.99, both ridge and KL estimators outperform the Liu estimator. None of the estimators uniformly dominates each other. However, it appears that our proposed estimator, KL, performs better in the wider space of *d* = *k* in the parameter space. If we review all Tables 1–8, we observed that the conclusions about the performance of all estimators remain the same for both *p*=3 and *p*=7.

## 4. Numerical Examples

To illustrate our theoretical results, we consider two datasets: (i) famous Portland cement data originally adopted by Woods et al. [34] and (ii) French economy data from Chatterjee and Hadi [35], and they are analyzed in the following sections, respectively.

### 4.1. Example 1: Portland Data

These data are widely known as the Portland cement dataset. It was originally adopted by Woods et al. [34]. It has also been analyzed by the following authors: Kaciranlar et al. [36]; Li and Yang [25]; and recently by Lukman et al. [13]. The regression model for these data is defined as

(46) $$\begin{array}{c} y_{i}=\beta_{0}+\beta_{1}X_{1}+\beta_{2}X_{2}+\beta_{3}X_{3}+\beta_{4}X_{4}+\varepsilon_{i}, \end{array}$$

where *y*_*i*_ = heat evolved after 180 days of curing measured in calories per gram of cement, *X*_1_ = tricalcium aluminate, *X*_2_ = tricalcium silicate, *X*_3_ = tetracalcium aluminoferrite, and *X*_4_ = *β*-dicalcium silicate. The correlation matrix of the predictor variables is given in Table 9.

The variance inflation factors are VIF_1_ = 38.50, VIF_2_ = 254.42, VIF_3_ = 46.87, and VIF_4_ = 282.51. Eigenvalues of *X*′*X* are *λ*_1_ = 44676.206, *λ*_2_ = 5965.422, *λ*_3_ = 809.952, and *λ*_4_ = 105.419, and the condition number of *X*′*X* is approximately 424. The VIFs, the eigenvalues, and the condition number all indicate the presence of severe multicollinearity. The estimated parameters and MSE are presented in Table 10. It appears from Table 11 that the proposed estimator performed the best in the sense of smaller MSE.

### 4.2. Example 2: French Economy Data

The French economy data in Chatterjee and Hadi [37] are considered in this example. It has been analyzed by Malinvard [38] and Liu [6], among others. The variables are imports, domestic production, stock formation, and domestic consumption. All are measured in milliards of French francs for the years 1949 through 1966.

The regression model for these data is defined as

(47) $$\begin{array}{c} y_{i}=\beta_{0}+\beta_{1}X_{1}+\beta_{2}X_{2}+\beta_{3}X_{3}+\varepsilon_{i}, \end{array}$$

where *y*_*i*_ = IMPORT, *X*_1_ = domestic production, *X*_2_ = stock formation, and *X*_3_ = domestic consumption. The correlation matrix of the predicted variable is given in Table 12.

The variance inflation factors are VIF_1_=469.688, VIF_2_=1.047, and VIF_3_=469.338. The eigenvalues of the *X*′*X* matrix are *λ*_1_ = 161779, *λ*_2_ = 158, and *λ*_3_ = 49.61, and the condition number is 32612. If we review the above correlation matrix, VIFs, and condition number, it can be said that there is presence of severe multicollinearity existing in the predictor variables.

The biasing parameter for the new estimator is defined in (41) and (42). The biasing parameter for the ridge and Liu estimator is provided in (6), (8), and (9), respectively.

We analyzed the data using the biasing parameters for each of the estimators and presented the results in Tables 10 and 11. It can be seen from Tables 10 and 11 that the proposed estimator performed the best in the sense of smaller MSE.

## 5. Summary and Concluding Remarks

In this paper, we introduced a new biased estimator to overcome the multicollinearity problem for the multiple linear regression model and provided the estimation technique of the biasing parameter. A simulation study has been conducted to compare the performance of the proposed estimator and Liu [6] and ridge regression estimators [3]. Simulation results evidently show that the proposed estimator performed better than both Liu and ridge under some condition on the shrinkage parameter. Two sets of real-life data are analyzed to illustrate the benefits of using the new estimator in the context of a linear regression model. The proposed estimator is recommended for researchers in this area. Its application can be extended to other regression models, for example, logistic regression, Poisson, ZIP, and related models, and those possibilities are under current investigation [37, 39, 40].

## Figures and Tables

**Table 1:** Estimated MSE when *n* = 30, *p*=3, and *ρ* = 0.70 and 0.80.

*n* = 30	0.7					0.8			
Sigma	*k* = *d*	OLS	Ridge	Liu	New est	OLS	Ridge	Liu	New est
1	0.1	0.362	0.352	0.291	0.342	0.547	0.519	0.375	0.491
	0.2		0.342	0.298	0.323		0.493	0.391	0.444
	0.3		0.333	0.305	0.307		0.470	0.407	0.404
	0.4		0.325	0.312	0.293		0.449	0.425	0.370
	0.5		0.317	0.320	0.280		0.431	0.443	0.342
	0.6		0.309	0.328	0.268		0.414	0.462	0.318
	0.7		0.302	0.336	0.258		0.398	0.482	0.299
	0.8		0.296	0.344	0.249		0.384	0.503	0.282
	0.9		0.290	0.353	0.242		0.372	0.525	0.269
	1.0		0.284	0.362	0.235		0.360	0.547	0.258

5	0.1	8.021	7.759	6.137	7.501	12.967	12.232	8.364	11.522
	0.2		7.511	6.331	7.021		11.567	8.817	10.261
	0.3		7.277	6.529	6.577		10.962	9.284	9.156
	0.4		7.056	6.731	6.165		10.411	9.766	8.186
	0.5		6.846	6.937	5.784		9.907	10.263	7.333
	0.6		6.647	7.146	5.430		9.445	10.775	6.581
	0.7		6.459	7.359	5.102		9.019	11.301	5.918
	0.8		6.280	7.576	4.797		8.626	11.842	5.331
	0.9		6.109	7.797	4.513		8.263	12.397	4.813
	1.0		5.947	8.021	4.250		7.926	12.967	4.354

10	0.1	31.993	30.939	24.421	29.907	51.819	48.871	33.333	46.022
	0.2		29.945	25.203	27.977		46.201	35.155	40.955
	0.3		29.005	26.000	26.189		43.775	37.034	36.514
	0.4		28.116	26.812	24.532		41.561	38.972	32.612
	0.5		27.274	27.639	22.995		39.536	40.968	29.176
	0.6		26.474	28.480	21.568		37.677	43.022	26.145
	0.7		25.715	29.336	20.241		35.966	45.134	23.466
	0.8		24.994	30.207	19.008		34.387	47.304	21.096
	0.9		24.307	31.092	17.860		32.926	49.532	18.996
	1.0		23.654	31.993	16.791		31.570	51.819	17.134

**Table 2:** Estimated MSE when *n* = 30, *p*=3, and *ρ* = 0.90 and 0.99.

*n* = 30	0.9					0.99			
Sigma	*k* = *d*	OLS	Ridge	Liu	New est	OLS	Ridge	Liu	New est
1	0.1	1.154	1.012	0.532	0.883	12.774	4.155	0.857	1.128
	0.2		0.899	0.583	0.691		2.339	1.388	1.946
	0.3		0.809	0.638	0.555		1.603	2.117	3.278
	0.4		0.736	0.697	0.459		1.214	3.045	4.416
	0.5		0.675	0.762	0.392		0.978	4.170	5.340
	0.6		0.625	0.831	0.346		0.821	5.495	6.097
	0.7		0.582	0.905	0.317		0.712	7.017	6.725
	0.8		0.545	0.983	0.299		0.631	8.738	7.255
	0.9		0.514	1.066	0.291		0.571	10.656	7.708
	1		0.487	1.154	0.289		0.524	12.774	8.100

5	0.1	28.461	24.840	12.067	21.501	**319.335**	102.389	17.451	23.383
	0.2		21.945	13.492	16.402		56.008	31.445	40.368
	0.3		19.588	15.017	12.625		36.978	50.327	71.447
	0.4		17.641	16.640	9.805		26.816	74.095	98.322
	0.5		16.010	18.362	7.690		20.580	102.751	120.269
	0.6		14.627	20.184	6.104		16.415	136.293	138.268
	0.7		13.442	22.105	4.917		13.467	174.723	153.240
	0.8		12.418	24.124	4.036		11.293	218.040	165.880
	0.9		11.526	26.243	3.393		9.637	266.244	176.695
	1		10.741	28.461	2.935		8.343	**319.335**	186.058

10	0.1	113.841	99.331	48.088	85.947	**1277.429**	409.249	69.149	92.868
	0.2		87.726	53.814	65.494		223.571	125.195	160.554
	0.3		78.277	59.935	50.326		147.369	200.793	284.749
	0.4		70.466	66.450	38.986		106.666	295.943	392.184
	0.5		63.919	73.361	30.469		81.687	410.644	479.940
	0.6		58.368	80.667	24.064		64.998	544.898	551.916
	0.7		53.612	88.368	19.262		53.189	698.703	611.794
	0.8		49.498	96.464	15.687		44.476	872.060	662.350
	0.9		45.910	104.955	13.064		37.839	1064.960	705.611
	1		42.758	113.841	11.182		32.655	**1277.429**	743.065

**Table 3:** Estimated MSE when *n* = 100, *p*=3, and *ρ* = 0.70 and 0.80.

*n* = 100	0.9					0.99			
Sigma	*k* = *d*	OLS	Ridge	Liu	New est	OLS	Ridge	Liu	New est
1	0.1	0.1124	0.1121	0.1105	0.1118	0.1492	0.1478	0.1396	0.1465
	0.2		0.1118	0.1107	0.1114		0.1465	0.1404	0.1441
	0.3		0.1116	0.1108	0.1110		0.1453	0.1414	0.1420
	0.4		0.1114	0.1110	0.1106		0.1442	0.1423	0.1401
	0.5		0.1112	0.1112	0.1104		0.1432	0.1434	0.1384
	0.6		0.1110	0.1114	0.1101		0.1422	0.1444	0.1369
	0.7		0.1108	0.1116	0.1100		0.1412	0.1455	0.1356
	0.8		0.1106	0.1119	0.1099		0.1403	0.1467	0.1345
	0.9		0.1105	0.1121	0.1099		0.1395	0.1479	0.1336
	1		0.1104	0.1124	0.1099		0.1387	0.1492	0.1328

5	0.1	2.0631	2.0452	1.9126	2.0274	3.2440	3.1954	2.8523	3.1472
	0.2		2.0276	1.9289	1.9924		3.1480	2.8942	3.0538
	0.3		2.0102	1.9454	1.9583		3.1019	2.9365	2.9638
	0.4		1.9932	1.9619	1.9249		3.0570	2.9793	2.8771
	0.5		1.9764	1.9785	1.8922		3.0133	3.0224	2.7934
	0.6		1.9599	1.9952	1.8603		2.9707	3.0659	2.7128
	0.7		1.9436	2.0121	1.8291		2.9291	3.1098	2.6350
	0.8		1.9276	2.0290	1.7986		2.8887	3.1542	2.5600
	0.9		1.9119	2.0460	1.7688		2.8492	3.1989	2.4876
	1		1.8964	2.0631	1.7396		2.8108	3.2440	2.4178

10	0.1	8.1632	8.0901	7.5481	8.0174	12.9200	12.7234	11.3344	12.5287
	0.2		8.0182	7.6150	7.8747		12.5320	11.5045	12.1511
	0.3		7.9474	7.6822	7.7351		12.3456	11.6761	11.7867
	0.4		7.8777	7.7498	7.5984		12.1640	11.8493	11.4349
	0.5		7.8091	7.8178	7.4646		11.9870	12.0239	11.0953
	0.6		7.7415	7.8862	7.3336		11.8144	12.2001	10.7674
	0.7		7.6750	7.9549	7.2053		11.6462	12.3778	10.4506
	0.8		7.6096	8.0240	7.0797		11.4821	12.5570	10.1447
	0.9		7.5451	8.0934	6.9568		11.3220	12.7377	9.8490
	1		7.4816	8.1632	6.8364		11.1658	12.9200	9.5634

**Table 4:** Estimated MSE when *n* = 100, *p*=3, and *ρ* = 0.90 and 0.99.

*n* = 30	0.9					0.99			
Sigma	*k* = *d*	OLS	Ridge	Liu	New est	OLS	Ridge	Liu	New est
1	0.1	0.287	0.278	0.230	0.270	3.072	2.141	0.688	1.423
	0.2		0.270	0.236	0.255		1.621	0.836	0.769
	0.3		0.263	0.241	0.242		1.298	1.013	0.528
	0.4		0.256	0.247	0.231		1.083	1.219	0.472
	0.5		0.250	0.253	0.221		0.930	1.455	0.506
	0.6		0.244	0.259	0.213		0.819	1.720	0.583
	0.7		0.239	0.265	0.206		0.733	2.014	0.680
	0.8		0.234	0.272	0.200		0.667	2.337	0.785
	0.9		0.230	0.279	0.195		0.613	2.690	0.892
	1		0.226	0.287	0.191		0.570	3.072	0.997

5	0.1	6.958	6.719	5.256	6.486	**76.772**	53.314	14.746	34.689
	0.2		6.495	5.431	6.050		39.905	18.971	16.660
	0.3		6.283	5.610	5.649		31.412	23.862	8.834
	0.4		6.083	5.792	5.278		25.626	29.420	5.803
	0.5		5.893	5.977	4.935		21.466	35.645	5.174
	0.6		5.714	6.166	4.617		18.350	42.537	5.795
	0.7		5.544	6.359	4.324		15.939	50.096	7.072
	0.8		5.383	6.555	4.052		14.024	58.321	8.686
	0.9		5.230	6.754	3.799		12.471	67.213	10.458
	1		5.085	6.958	3.566		11.189	**76.772**	12.287

10	0.1	27.809	26.853	20.970	25.916	307.086	213.255	58.717	138.685
	0.2		25.951	21.675	24.167		159.582	75.683	66.354
	0.3		25.100	22.394	22.551		125.559	95.308	34.815
	0.4		24.296	23.126	21.056		102.365	117.590	22.463
	0.5		23.535	23.872	19.672		85.681	142.529	19.743
	0.6		22.815	24.632	18.389		73.175	170.126	22.045
	0.7		22.131	25.406	17.200		63.493	200.380	26.995
	0.8		21.482	26.193	16.096		55.802	233.291	33.308
	0.9		20.865	26.994	15.071		49.561	268.860	40.270
	1		20.279	27.809	14.120		44.407	**307.086**	47.470

**Table 5:** Estimated MSE when *n* = 30, *p*=7, and *ρ* = 0.70 and 0.80.

*n* = 30	0.7					0.8			
Sigma	*k* = *d*	OLS	Ridge	Liu	New ridge	OLS	Ridge	Liu	New ridge
1	0.1	0.838	0.811	0.651	0.785	1.239	1.179	0.859	1.121
	0.2		0.786	0.670	0.737		1.124	0.895	1.018
	0.3		0.763	0.689	0.694		1.074	0.933	0.928
	0.4		0.741	0.709	0.654		1.029	0.973	0.850
	0.5		0.720	0.729	0.618		0.987	1.014	0.781
	0.6		0.701	0.750	0.586		0.949	1.056	0.721
	0.7		0.682	0.771	0.556		0.914	1.100	0.669
	0.8		0.665	0.793	0.529		0.881	1.145	0.623
	0.9		0.649	0.815	0.505		0.851	1.191	0.583
	1		0.633	0.838	0.484		0.823	1.239	0.549

5	0.1	20.955	20.275	16.063	19.608	30.981	29.455	21.084	27.975
	0.2		19.633	16.568	18.362		28.060	22.071	25.314
	0.3		19.026	17.083	17.208		26.780	23.086	22.951
	0.4		18.452	17.607	16.139		25.602	24.130	20.845
	0.5		17.908	18.141	15.147		24.513	25.201	18.963
	0.6		17.391	18.685	14.226		23.506	26.301	17.279
	0.7		16.901	19.238	13.369		22.570	27.429	15.767
	0.8		16.435	19.801	12.572		21.699	28.585	14.408
	0.9		15.990	20.373	11.829		20.885	29.769	13.185
	1		15.567	20.955	11.137		20.125	30.981	12.081

10	0.1	83.821	81.095	64.205	78.423	123.923	117.811	84.259	111.887
	0.2		78.523	66.233	73.429		112.224	88.219	101.225
	0.3		76.091	68.299	68.804		107.097	92.291	91.749
	0.4		73.789	70.403	64.513		102.375	96.475	83.301
	0.5		71.608	72.545	60.530		98.014	100.770	75.750
	0.6		69.537	74.725	56.827		93.973	105.177	68.983
	0.7		67.569	76.942	53.382		90.220	109.696	62.908
	0.8		65.698	79.197	50.173		86.725	114.327	57.441
	0.9		63.915	81.490	47.182		83.463	119.069	52.515
	1		62.215	83.821	44.392		80.411	123.923	48.069

**Table 6:** Estimated MSE when *n* = 30, *p*=7, and *ρ* = 0.9 and 0.99.

*N* = 30	0.9					0.99			
Sigma	*k* = *d*	OLS	Ridge	Liu	New ridge	OLS	Ridge	Liu	New ridge
1	0.1	2.52	2.27	1.29	2.03	28.68	11.20	2.26	4.45
	0.2		2.06	1.39	1.66		6.82	3.55	4.16
	0.3		1.88	1.51	1.37		4.78	5.25	5.78
	0.4		1.73	1.63	1.16		3.62	7.36	7.58
	0.5		1.61	1.76	0.99		2.88	9.89	9.25
	0.6		1.50	1.90	0.85		2.37	12.83	10.75
	0.7		1.41	2.04	0.75		2.01	16.17	12.07
	0.8		1.32	2.20	0.68		1.74	19.93	13.24
	0.9		1.25	2.35	0.62		1.54	24.10	14.27
	1		1.18	2.52	0.57		1.38	28.68	15.19

5	0.1	63.03	56.58	31.23	50.57	717.09	278.85	50.83	108.00
	0.2		51.27	34.11	41.03		168.23	84.11	97.17
	0.3		46.82	37.15	33.61		116.38	127.57	134.54
	0.4		43.03	40.35	27.78		86.52	181.23	176.89
	0.5		39.77	43.72	23.14		67.36	245.07	216.47
	0.6		36.94	47.25	19.42		54.18	319.10	251.87
	0.7		34.45	50.95	16.43		44.67	403.32	283.16
	0.8		32.25	54.81	14.01		37.54	497.72	310.80
	0.9		30.28	58.84	12.06		32.06	602.31	335.29
	1		28.52	63.03	10.48		27.73	717.09	357.10

10	0.1	252.14	226.30	124.75	202.23	2868.35	1115.06	202.39	431.48
	0.2		205.03	136.28	164.03		672.43	335.62	387.84
	0.3		187.21	148.46	134.32		464.91	509.59	537.07
	0.4		172.05	161.30	110.91		345.38	724.31	706.26
	0.5		158.99	174.79	92.29		268.65	979.78	864.43
	0.6		147.63	188.95	77.37		215.88	1276.00	1005.86
	0.7		137.66	203.76	65.34		177.77	1612.96	1130.89
	0.8		128.82	219.23	55.62		149.24	1990.68	1241.34
	0.9		120.95	235.35	47.75		127.25	2409.14	1339.21
	1		113.89	252.14	41.38		109.92	2868.35	1426.34

**Table 7:** Estimated MSE when *n* = 100, *p*=7, and *ρ* = 0.70 and 0.80.

*n* = 100	0.7					0.8			
Sigma	*k* = *d*	OLS	Ridge	Liu	New ridge	OLS	Ridge	Liu	New ridge
1	0.1	0.174	0.173	0.163	0.171	0.263	0.259	0.235	0.255
	0.2		0.171	0.164	0.169		0.255	0.238	0.249
	0.3		0.170	0.165	0.166		0.252	0.241	0.243
	0.4		0.169	0.166	0.164		0.249	0.244	0.237
	0.5		0.167	0.168	0.161		0.246	0.247	0.232
	0.6		0.166	0.169	0.159		0.243	0.250	0.227
	0.7		0.165	0.170	0.157		0.240	0.253	0.222
	0.8		0.164	0.171	0.155		0.238	0.256	0.218
	0.9		0.163	0.173	0.154		0.235	0.259	0.214
	1		0.162	0.174	0.152		0.233	0.263	0.210

5	0.1	4.356	4.320	4.055	4.284	6.563	6.474	5.852	6.386
	0.2		4.285	4.087	4.214		6.388	5.928	6.216
	0.3		4.250	4.120	4.146		6.304	6.005	6.053
	0.4		4.216	4.153	4.079		6.222	6.082	5.895
	0.5		4.182	4.187	4.013		6.143	6.160	5.744
	0.6		4.149	4.220	3.949		6.066	6.239	5.598
	0.7		4.116	4.254	3.887		5.991	6.319	5.457
	0.8		4.084	4.288	3.826		5.917	6.399	5.322
	0.9		4.053	4.322	3.767		5.846	6.481	5.191
	1		4.022	4.356	3.708		5.777	6.563	5.066

10	0.1	17.425	17.281	16.219	17.138	26.250	25.896	23.408	25.545
	0.2		17.140	16.350	16.858		25.551	23.713	24.866
	0.3		17.001	16.482	16.584		25.216	24.020	24.212
	0.4		16.864	16.614	16.316		24.891	24.330	23.582
	0.5		16.729	16.748	16.054		24.573	24.643	22.975
	0.6		16.597	16.882	15.797		24.265	24.959	22.389
	0.7		16.467	17.016	15.547		23.964	25.277	21.825
	0.8		16.339	17.152	15.301		23.671	25.599	21.280
	0.9		16.213	17.288	15.062		23.385	25.923	20.755
	1		16.089	17.425	14.827		23.107	26.250	20.247

**Table 8:** Estimated MSE when *n* = 100, *p*=7, and *ρ* = 0.90 and 0.99.

*n* = 100	0.9					0.99			
Sigma	*k* = *d*	OLS	Ridge	Liu	New ridge	OLS	Ridge	Liu	New ridge
1	0.1	0.546	0.529	0.431	0.512	6.389	4.391	1.624	2.949
	0.2		0.513	0.442	0.482		3.407	1.934	1.836
	0.3		0.498	0.454	0.456		2.819	2.298	1.453
	0.4		0.485	0.466	0.432		2.423	2.718	1.347
	0.5		0.472	0.478	0.411		2.135	3.192	1.359
	0.6		0.460	0.491	0.392		1.914	3.721	1.426
	0.7		0.449	0.504	0.375		1.738	4.306	1.519
	0.8		0.439	0.517	0.360		1.593	4.945	1.625
	0.9		0.429	0.531	0.346		1.472	5.640	1.737
	1		0.420	0.546	0.334		1.370	6.389	1.851

5	0.1	13.640	13.216	10.676	12.802	159.732	109.722	38.895	73.284
	0.2		12.820	10.979	12.037		84.915	47.018	44.506
	0.3		12.448	11.289	11.336		69.971	56.467	33.865
	0.4		12.099	11.605	10.693		59.823	67.242	30.146
	0.5		11.770	11.928	10.102		52.370	79.343	29.417
	0.6		11.460	12.257	9.558		46.597	92.769	30.090
	0.7		11.168	12.593	9.056		41.953	107.521	31.455
	0.8		10.891	12.935	8.593		38.114	123.599	33.171
	0.9		10.628	13.284	8.165		34.875	141.003	35.063
	1		10.379	13.640	7.768		32.097	159.732	37.036

10	0.1	54.558	52.866	42.699	51.212	638.928	438.910	155.399	293.121
	0.2		51.282	43.914	48.150		339.663	187.945	177.874
	0.3		49.796	45.155	45.344		279.860	225.785	135.151
	0.4		48.399	46.422	42.768		239.236	268.921	120.120
	0.5		47.084	47.714	40.397		209.391	317.351	117.053
	0.6		45.843	49.032	38.214		186.265	371.077	119.599
	0.7		44.670	50.375	36.198		167.659	430.097	124.922
	0.8		43.560	51.744	34.336		152.274	494.412	131.654
	0.9		42.508	53.138	32.612		139.287	564.022	139.094
	1		41.509	54.558	31.014		128.149	638.928	146.866

**Table 9:** Correlation matrix.

	*X*_1_	*X*_2_	*X*_3_	*X*_4_
*X*_1_	1.000	0.229	−0.824	−0.245
*X*_2_	0.229	1.000	−0.139	−0.973
*X*_3_	−0.824	−0.139	1.000	0.030
*X*_4_	−0.245	−0.973	0.030	1.000

**Table 10:** The results of regression coefficients and the corresponding MSE values.

Coef.	$\hat{\alpha }$	$\hat{\alpha }\left(k\right)$	$\hat{\alpha }\left(d\right)$ ${\hat{d}}_{\text{alt}}$	${\hat{\alpha }}_{\text{KL}}\left(k_{\text{HNM}}\right)$	${\hat{\alpha }}_{\text{KL}}\left(k_{\min}\right)$
*α*_0_	62.4054	8.5870	27.6490	−19.7876	27.6068
*α*_1_	1.5511	2.1046	1.9010	2.3965	1.9090
*α*_2_	0.5102	1.0648	0.8701	1.3573	0.8688
*α*_3_	0.1019	0.6681	0.4621	0.9666	0.4680
*α*_4_	−0.1441	0.3996	0.2082	0.6862	0.2074
MSE	4912.09	2989.83	2170.963	7255.603	2170.96
*k*/*d*	—	0.0077	0.44195	0.00235	0.00047

**Table 11:** The results of regression coefficients and the corresponding MSE values.

Coef.	$\hat{\alpha }$	$\hat{\alpha }\left(k\right)$	$\hat{\alpha }\left(d\right)$ ${\hat{d}}_{\text{alt}}$	$\hat{\alpha }\left(d\right){\hat{d}}_{\text{opt}}$	${\hat{\alpha }}_{\text{KL}}\left(k_{\text{HNM}}\right)$	${\hat{\alpha }}_{\text{KL}}\left(k_{\min}\right)$
*α*_0_	−19.7127	−16.7613	−12.5762	−18.8410	−16.5855	−18.8782
*α*_1_	0.0327	0.1419	0.2951	0.0648	0.1485	0.0636
*α*_2_	0.4059	0.3576	0.2875	0.3914	0.3548	0.3922
*α*_3_	0.2421	0.0709	−0.1696	0.1918	0.0606	0.1937
MSE	17.3326	21.30519	58.28312	16.60293	22.11899	16.60168
*k*/*d*	—	0.0527	0.5282	0.9423	0.0258	0.0065

**Table 12:** Correlation matrix.

	*X*_1_	*X*_2_	*X*_3_
*X*_1_	1.000	0.210	0.999
*X*_2_	0.210	1.000	0.208
*X*_3_	0.999	0.208	1.000

## Data Availability

Data will be made available on request.
